# Exon 1 Disruption Alters Tissue-Specific Expression of Mouse p53 and Results in Selective Development of B Cell Lymphomas

**DOI:** 10.1371/journal.pone.0049305

**Published:** 2012-11-14

**Authors:** Y. Jeffrey Chiang, Michael J. Difilippantonio, Lino Tessarollo, Herbert C. Morse, Richard J. Hodes

**Affiliations:** 1 Experimental Immunology Branch, National Cancer Institute, National Institutes of Health, Bethesda, Maryland, United States of America; 2 Genetics Branch, Center for Cancer Research, National Cancer Institute, National Institutes of Health, Bethesda, Maryland, United States of America; 3 Center for Cancer Research, Mouse Cancer Genetics Program, National Cancer Institute, Frederick, Maryland, United States of America; 4 Laboratory of Immunogenetics, National Institute of Allergy and Infectious Diseases, National Institutes of Health, Rockville, Maryland, United States of America; 5 National Institute on Aging, National Institutes of Health, Bethesda, Maryland, United States of America; University of Miami, United States of America

## Abstract

p53 is a tumor suppressor gene mutated in >50% of human cancers, while p53 deficiency in mice results in cancers and accelerated mortality. Thymic T cell lymphoma is the most common malignancy in p53-deficient mice, making it difficult to study the role of p53 in other malignancies. To overcome this limitation, we attempted to generate mice with a reversible p53 knockout (p53^rev/rev^) by inserting a floxed transcriptional stop into the first exon of p53, anticipating that this would allow tissue-specific Cre-mediated expression of p53. Contrary to expectations, functional p53 protein was expressed in the thymus and multiple other tissues of p53^rev/rev^ mice in the absence of Cre, whereas B cells expressed p53 protein only in the presence of B cell-specific CD19-Cre. In the absence of Cre, 76% of p53^rev/rev^ mice developed splenic marginal zone B cell lymphomas, indicating sensitivity of this B cell subset to transformation caused by p53 deficiency. 5′-RACE identified p53 mRNA transcribed from a novel start site utilized in thymocytes but not normal B cells or B cell lymphomas from p53^rev/rev^ mice. The p53^rev/rev^ mouse thus demonstrates an effect of p53 deficiency in development of splenic marginal zone lymphomas and provides a model for study of p53-deficient human B cell lymphomas.

## Introduction

The tumor suppressor gene, *Trp53*, which encodes the p53 protein, plays an important role in maintaining genomic integrity in response to a wide range of cellular stresses including DNA damage, hypoxia, ribonucleotide depletion, and oncogene activation [1]. These stress signals stimulate the activation of p53 protein, resulting in effects on multiple cellular processes including apoptosis, cell cycle arrest, and senescence, mediated largely through the activity of p53 in transcriptional regulation of its target genes [2], [3], [4]. Dysfunction of p53 can predispose to the development or progression of cancers. In fact, the p53 gene is the most frequently mutated gene identified in a variety of human cancers with more than 50% of human tumors characterized by p53 mutations [5]. Further evidence for a critical tumor suppressor role of p53 is provided by analysis of p53 knockout mice (p53^−/−^), which develop tumors, predominantly thymic T cell lymphomas, at an early age in essentially 100% of homozygous p53-deficient mice [6], [7], [8].

p53^−/−^ mice, deficient for expression of all p53 isoforms, are developmentally normal but are susceptible to a variety of spontaneous tumors by about 6 months of age [6], [9]. About 75% of the tumors that develop in p53^−/−^ mice on either C57BL/6 or 129/Sv genetic backgrounds are T-cell lymphomas [10]. Sarcomas also develop in p53 knockout mice [9], [10], and other malignancies, such as B-cell lymphomas, occur less frequently [9], [10]. To assess the role of p53 in the initiation and progression of other malignancies, we attempted to generate a reversible p53 knockout mouse (p53^rev/rev^) by inserting a neomycin resistance gene flanked by loxP sites into the first exon of the p53 gene. Based on current understandings of p53 transcription, it was anticipated that, in the absence of Cre-mediated recombination, this would introduce a transcriptional stop signal, so that p53^rev/rev^ mice would not express p53 protein and would develop tumors precisely as observed in conventional p53^−/−^ mice. Surprisingly, however, expression of p53 protein was detected at varying levels in multiple tissues of p53^rev/rev^ mice, with expression level in thymus similar to that of wild-type (wt) mice, but with reduced levels in spleen, uterus, kidney, liver and heart, and with no detectable p53 in peripheral B lymphocytes. Analysis by 5′RACE revealed that thymi of p53^rev/rev^ mice expresses a new species of p53 mRNA transcribed from a novel start site utilized in thymocytes but not in p53^rev/rev^ B cells. p53^rev/rev^ mice developed splenic B cell lymphomas at high frequency, but did not develop the thymic lymphomas that are characteristic of p53^−/−^ mice.

## Materials and Methods

### Mice

p53^+/−^ mice [11] and CD19-Cre^+^ transgenic mice [12] were purchased from the Jackson Laboratory (Bar Harbor, ME). p53^+/−^ mice were intercrossed to produce p53^−/−^ mice. p53^rev/rev^ mice, generated as described below, were backcrossed with C57BL/6 (B6) mice for at least six generations. Protocols for animal care and use were conducted consistent with the Guide for the Animal Care and Use of Laboratory Animals of National Institutes of Health. The protocol was approved by the Committee of the Animal Care and Use of Laboratory Animals of National Institutes of Health (IACUC protocol number: EIB-029). No surgery was performed, and humane endpoints were followed per IACUC guidelines. All animals were housed at Bioqual (Rockville, MD) and experiments were performed in NCI.

### Generation of Trp53 gene targeted mice

**Figure 1:**
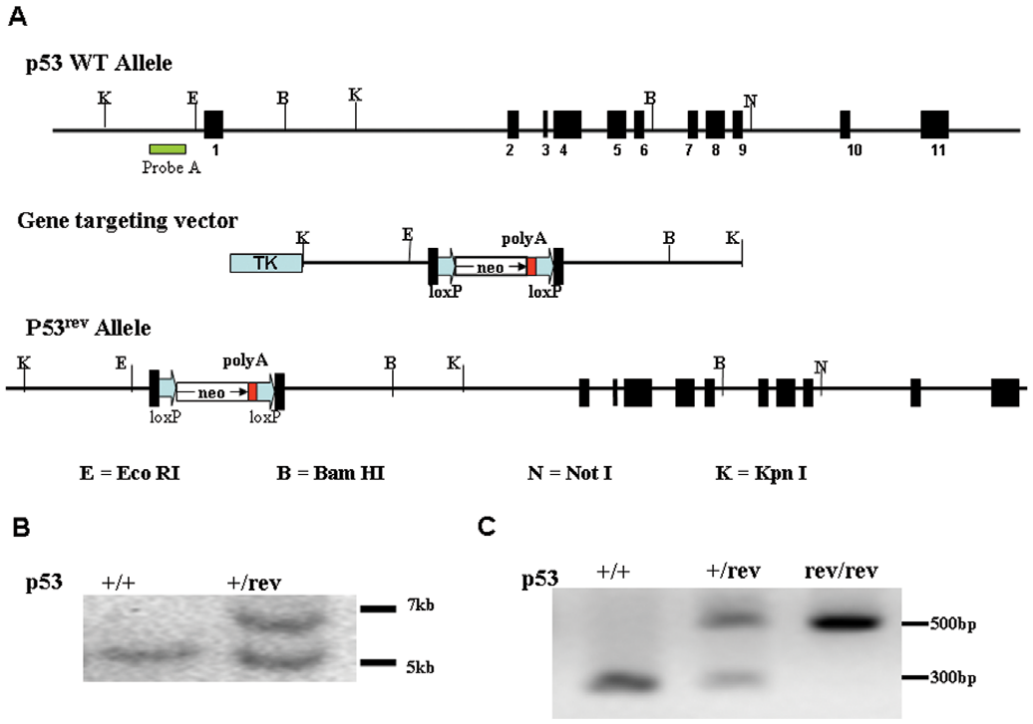
Generation of p53^rev/rev^ mice. A) Gene targeting strategy and restriction map of the p53 gene. Filled boxes indicate exons; labeled boxes indicate neomycin resistance or herpesvirus thymidine kinase (tk) genes; and arrows indicate loxP sites. B) Southern blot analysis of ES cell DNA. The 5 kb band represents the germ line allele and 7 kb band represents the targeted allele after Kpn I digestion and hybridization with probe A. C) PCR analysis for the p53^rev/rev^ mouse genotype. The 300 and 500-bp PCR products represent the wt and gene-targeted alleles, respectively.

A *Trp53* gene targeting vector was constructed from a 5 kb DNA segment including exon 1 of the *Trp53 gene*, and was isolated from 129 mouse genomic DNA by digestion with Kpn I. A neomycin resistance cassette flanked by loxP sites was inserted into the first exon of the gene (Figure 1A). A thymidine kinase cassette (TK) was placed further upstream of exon 1 and used as a negative selection marker. Electroporation and selection were performed with the 129 CJ7 embryonic stem (ES) cell line as described by Southon and Tessarollo [13]. Two independent successfully targeted ES cell clones were injected into C57BL/6CR blastocysts, generating chimeras that transmitted the targeted allele to progeny.

### Genotyping for p53^rev/rev^ ES cells and mice with Southern blot

DNA for Southern blot analysis was isolated from ES cells. DNA isolation and Southern blot analysis procedures were as described elsewhere [14], [15]. DNA was digested with Kpn I, electrophoresed on a 0.7% agarose gel, transferred to a nylon membrane, and hybridized with probe A (Fig. 1A.)

### Reverse transcription PCR (RT-PCR)

A NucleoSpin kit (Clontech, Mountain View, CA) was used to isolate total RNA from various tissues or cells for RT-PCR analysis. Primer pair E1 (5′-TTA GGG GGC ACC TAG CAT TC-3′) and E4 (5′-AGT TGC CCT GGT AAG TTT TTT G-3′) was used to detect conventional p53 mRNA. Primer pair N2F (GACGTA AAC TCC TCT TCA G) and E4 was used to detect the expression of new species of p53 mRNA. The primer pair N1F (GGG CGC CCG GTT CTT TTT GTC A) and N1R (TTG GTG GTC GAA TGG GCA GGT AGC) amplifies transcripts from the neomycin resistance gene. Actin primer pair ACTIN-F (5′-ATG CCA ACA CAG TGC TGT CTG GTG G-3′) and ACTIN-R (5′-CTG ATC CAC ATC TGC TGG AAG GTG-3′) was used to detect the expression of β-actin. RT-PCR reactions were carried out in 50 µl of PCR reaction mix containing 25 µl of PCR buffer (One-step RT-PCR kit, Life Technologies), 1 µg of total RNA and 0.2 µM primers. RT-PCR was carried out at 42°C for 30 minutes, 35 cycles of 95°C for 30 sec, 58°C for 30 sec and 72°C for 1 min. RT-PCR products were visualized by agarose gel electrophoresis with EtBr staining.

### 5′Rapid amplification of mRNA end by PCR (5′-RACE)

5′-RACE was performed according to instructions for the 5′-RACE kit purchased from Invitrogen (San Diego, CA). Primer p53-1165R (ACC ATC ATC ACA CTG GAA GAC) was used to make cDNA. The primer pair adaptor primer and p53-411R (AGT TGC CCT GGT AAG TTT TTT G) was used to amplify the 5′ end of the single stranded cDNA ligated with adaptor, and PCR products were sequenced.

### Western blot analysis for p53 protein

Mice or cells were irradiated at 10 Gy. Three hours later, mouse tissues or cells were lysed in buffer containing 50 µm Tris (pH 7.4), 150 µm NaCl, 1 mM Na_2_VO_4_, 1%NP-40, and protease inhibitor cocktail. Twenty-five micrograms of protein extract from each sample was fractionated by sodium dodecyl sulfate-polyacrylamide gel electrophoresis and transferred to a nitrocellulose membrane. Immunoblotting analysis was carried out with monoclonal antibodies specific for p53 (1C12) (Cell Signaling, Beverly, MA) [15] and actin (AC-15) (Sigma, St. Louis, MO).

### Apoptosis analysis for thymocytes and splenocytes in response to ionizing radiation

Thymocytes and splenocytes were treated with ionizing radiation at 10 Gy, cultured at 37°C for 24 hours and stained with PI and annexin V-FITC for flow cytometric (FACS) analysis [16], [17].

### Histological analysis

Tumors were fixed in 4% paraformaldehyde at 4°C overnight, dehydrated through a graded alcohol series, and then embedded in paraffin. Sections of 6–8 µm were prepared and stained with H&E [14]. Tumors were classified according to established criteria [18].

### Analysis of chromosomal aberrations by Spectral Karyotyping (SKY)

Metaphase spreads were prepared and Spectral Karyotyping (SKY) was performed for the identification of chromosomal abnormalities according to standard protocols [19] available at http://www.riedlab.nci.nih.gov/protocols.asp. A minimum of 10 metaphases were acquired and analyzed for each cell line-derived tumor. The karyotypic findings are described in accordance with the ISCN nomenclature rules (ISCN, 2005).

## Results

### Generation of mice bearing a p53 reversible knockout gene

p53 reversible knockout (*p53^rev/rev^*) mice were generated with the intent of allowing cell type-specific expression of p53. The first exon of p53 was disrupted by inserting a neomycin resistance cassette (neo) flanked with loxP sites (Figure 1A). It was anticipated that insertion of the neo cassette would result in termination of p53 transcription with the polyA signal sequence in the neo cassette before the transcript elongates into intron 1. The gene-targeting vector was transfected into ES cells, and Southern blot analysis was used to identify gene targeted ES cell clones. Wild-type ES cell DNA generated a 5 kb band with probe A (Figure 1A) after Kpn I digestion, while heterozygous (p53^+/rev^) ES cell DNA generated one additional 7 kb band (Figure 1B). Four of 85 ES cell clones tested were identified as containing the p53^rev^ allele. Two of the positive clones were used to make chimeric mice that transmitted the p53^rev^ allele to offspring. These mice were backcrossed to B6 background for at least 6 generations. p53^+/rev^ mice were intercrossed to produce p53^rev/rev^ mice as genotyped by PCR (Figure 1C). Development and early survival of p53^rev/rev^ mice were normal, with 21 p53^+/+^, 40 p53^+/rev^, and 19 p53^rev/rev^ mice observed at weaning among 80 offspring from p53^+/rev^ intercrosses, consistent with Mendelian segregation and indicating that no early lethality was associated with the p53^rev/rev^ genotype.

### Differential expression of p53 protein in tissues and cell lineages of p53^rev/rev^ mice: detection in thymocytes but not peripheral B lymphocytes

**Figure 2:**
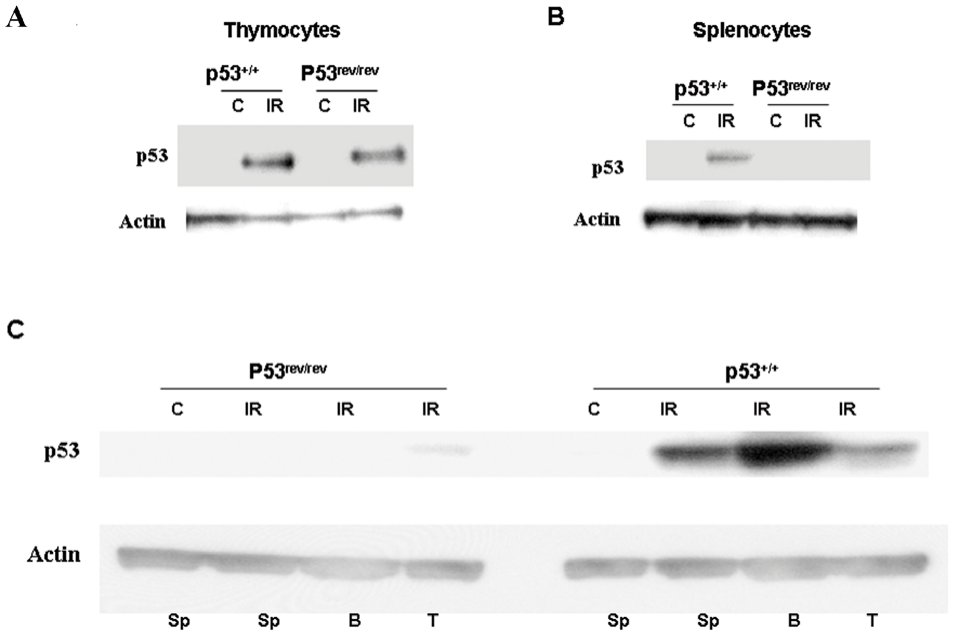
Expression of p53 in lymphocytes of p53^rev/rev^ mice. A) Western blot analysis was used to determine p53 protein expression in thymocytes of wt and p53^rev/rev^ mice as indicated. Actin protein expression was used as a loading control. The results shown are representative of four independent experiments. B) Western blot analysis was used to determine p53 protein expression in spleen cells of wt and p53^rev/rev^ mice as indicated. Actin protein expression was used as a loading control. The results shown are representative of four independent experiments. C) Western blot analysis was used to determine p53 protein expression in total spleen cells (SP), purified splenic B cells (B) and purified splenic T cells (T) of p53^+/+^ and p53^rev/rev^ mice with (IR) or without (C) irradiation as indicated. Actin protein expression was used as a loading control. The results shown are representative of two independent experiments.

It was anticipated that no p53 protein would be produced in p53^rev/rev^ mice in the absence of Cre-mediated recombination. Normally, p53 protein is expressed but not easily detected due to its rapid proteasomal degradation under steady-state conditions [2], [20]. To assess p53 protein expression in p53^rev/rev^ and wt controls, mice were irradiated at 10 Gy to increase p53 protein levels, largely through post-translational stabilization [21]. Tissues taken from these mice 3 hr later were used to make protein lysates that were analyzed by western blotting with p53-specific antibodies. Surprisingly, p53 protein was detected in multiple tissues in both wt and p53^rev/rev^ mice with the notable exception that its level was markedly reduced in spleen of p53^rev/rev^ mouse (Figure S1). We then proceeded to study isolated populations of thymocytes and spleen cells for the effects of radiation on p53 expression in vitro. As shown in Figure 2A, comparable levels of p53 protein were induced in response to irradiation of wt and p53^rev/rev^ thymocytes. In contrast, p53 protein was at or below the limits of detection in p53^rev/rev^ spleen cells but was readily detected in lysates from spleen cells of wt mice (Figure 2B). This prompted us to test purified splenic T cells and B cells for their responses to irradiation. Studies of cells from wt mice revealed striking elevations of p53 levels in irradiated B cells and lesser but still substantial elevations of expression in treated T cells (Figure 2C). By comparison, there was no detectable p53 response by B cells from p53^rev/rev^ mice and the response of treated T cells, while detectable, was considerably reduced from that of irradiated wt T cells.

### Characterization of p53 mRNA: Identification of a novel transcript in thymocytes from p53^rev/rev^ mice

**Figure 3:**
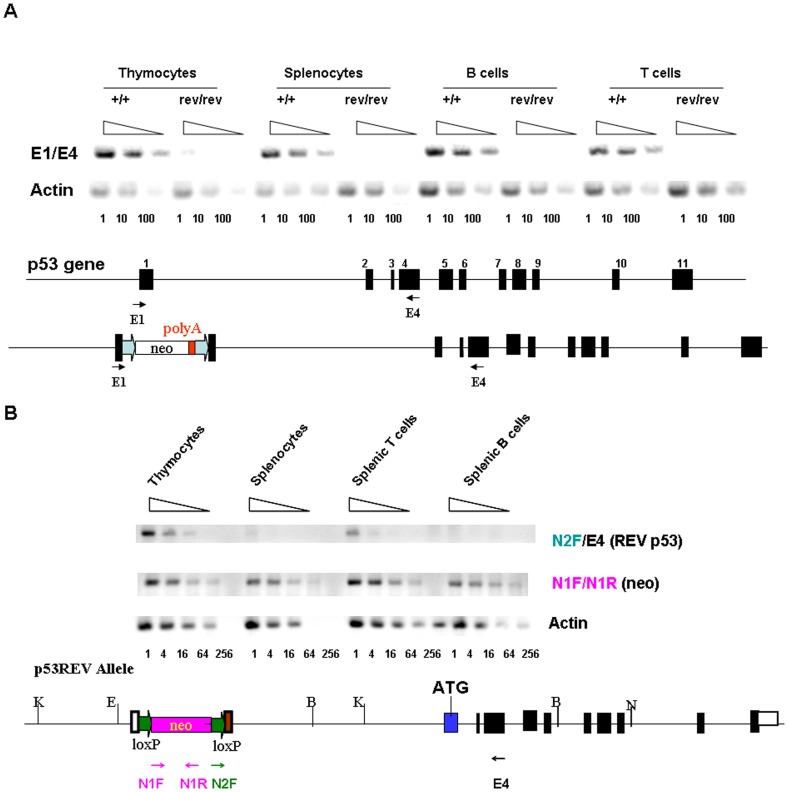
The expression of p53 mRNA and p53REV mRNA in thymocytes and splenocytes. A) RT-PCR analysis for p53 mRNA expression in thymocytes, splenocytes, spleen B cells and spleen T cells of wt and p53^rev/rev^ mice as indicated. Actin was used as a loading control. The results shown are representative of three independent experiments. The primers are shown relative to their position in the p53 gene. The numbers at the bottom indicate the dilution of RNA templates. B) RT-PCR analysis for p53REV and neomycin mRNA expression in thymocytes, spleen cells, splenic T cells and splenic B cells of p53^rev/rev^ mice as indicated. Actin was used as a loading control. The results shown are representative of two independent experiments. The primers are shown relative to their positions in p53REV and neomycin genes. E4 is also the RACE start site. The numbers at the bottom indicate the dilution of RNA templates. Filled boxes indicate exons; labeled boxes indicate neomycin resistance or herpesvirus thymidine kinase (tk) genes; and arrows indicate loxP sites.

To determine the mechanisms responsible for the unexpected expression of p53 protein in cells of p53^rev/rev^ mice, we first examined the nature of p53 transcripts by RT-PCR using primers from exon 1 and exon 4 (Figure 3A). p53 transcripts were readily detected in both thymocytes and spleen cells of wt mice, but were very low in thymocytes and not detectable in spleen cells of p53^rev/rev^ mice. We then used 5′-RACE to determine if thymocytes from p53^rev/rev^ mice might express alternative species of p53 transcripts that could be translated into p53 and identified a novel species of p53 mRNA, designated as p53REVmRNA (Figure S2). The 5′-RACE PCR product was sequenced in its entirety and found to include a 176 bp sequence at the 5′ end identical to the 3′-sequence of the neomycin resistance cassette that was used for gene targeting and that included the loxP site. This was followed by a 25 bp sequence identical to the 3′end of exon 1, and then exons 2 and 3 and part of exon 4. The sequences downstream from exon 4 were identical to the sequences of wt p53 cDNA. The p53 protein predicted to be translated from p53REV mRNA would thus be identical to wt p53. Figure S2 illustrates the new transcriptional start site of p53REV RNA and the approach taken for RT-PCR analysis of this transcript. RT-PCR analyses using primers E4 and N2F corresponding to a unique sequence in p53REV, revealed that p53REV mRNA is expressed in thymocytes and splenic T cells but not in splenic B cells of p53^rev/rev^ mice (Figure 3B), paralleling the patterns of p53 protein expression in thymocytes and spleen cells of these mice. In contrast, expression of the gene encoding the neomycin resistance protein, detected using primers N1F and N1R (Figure 3B), was comparable in both thymocytes and splenic B cells of p53^rev/rev^ mice, indicating that the expression of p53REV mRNA was not regulated by the promoter of the neomycin gene. The interpretation of these findings for regulation of p53 expression in p53^rev/rev^ mice is discussed below.

### Irradiation-induced p53-dependent apoptosis is intact in thymocytes but not spleen cells of p53^rev/rev^ mice

**Figure 4:**
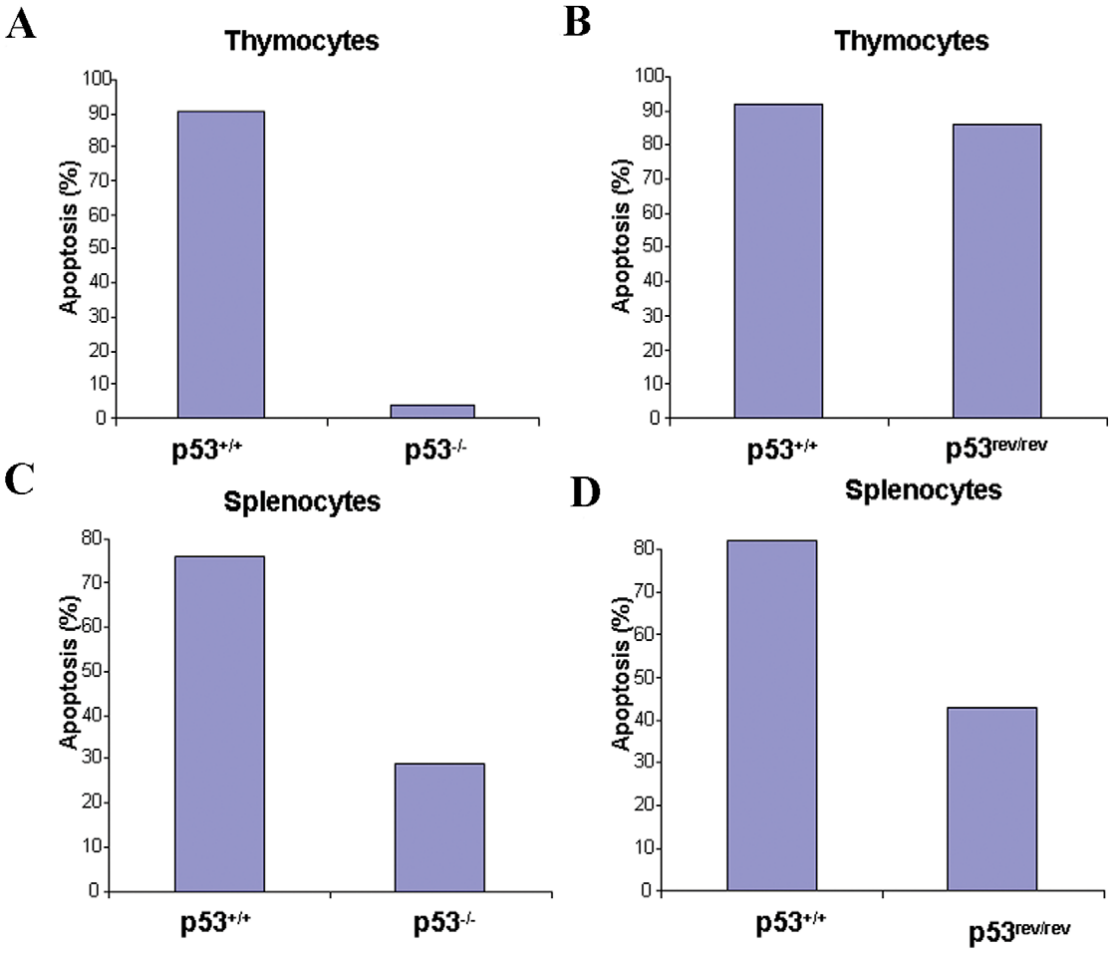
Apoptosis of thymocytes and splenocytes in response to irradiation. A) Apoptosis of littermate p53^−/−^ and p53^+/+^ thymocytes after irradiation and overnight culture. The results shown are representative of three independent experiments. B) Apoptosis of littermate p53^rev/rev^ and wt thymocytes after irradiation and overnight culture. The results shown are representative of three independent experiments. C) Apoptosis of littermate p53^−/−^ and p53^+/+^ spleen cells after irradiation and overnight culture. The results shown are representative of two independent experiments. D) Apoptosis of littermate p53^rev/rev^ and wt spleen cells after irradiation and overnight culture. The results shown are representative of two independent experiments.

A major function of p53 expressed in response to stress is the induction of apoptosis [2], [3], [20]. To compare the responses of cells from p53^rev/rev^ and wt mice to stress, thymocytes and splenocytes were irradiated and examined by FACS for apoptosis using analyses of Annexin V and PI staining. After irradiation and overnight culture, only 4% of thymocytes from p53^−/−^ mice had undergone apoptotic death in comparison to 91% of thymocytes from wt mice (Figure 4A), demonstrating the p53 dependence of radiation-induced apoptosis. Under the same conditions, similar proportions of thymocytes from wt and p53^rev/rev^ mice had undergone apoptosis (92% and 86%, respectively) (Figure 4B). These results indicated that thymocytes from p53^rev/rev^ mice that express p53 protein encoded by p53REV mRNA were as susceptible to radiation-induced apoptosis as thymocytes from mice expressing p53 from the wt transcript. Similar analyses of spleen cells showed that 29% of cells from p53^−/−^ mice underwent apoptosis post irradiation compared to 76% of cells from wt littermates (Figure 4C) whereas the proportions of apoptotic cells from p53^rev/rev^ and wt mice were 43% and 82%, respectively (Figure 4D). Thus, spleen cells from mice totally deficient in p53 and from p53^rev/rev^ mice were similarly resistant to radiation-induced apoptosis.

### p53^rev/rev^ mice spontaneously develop B cell lymphomas

**Figure 5:**
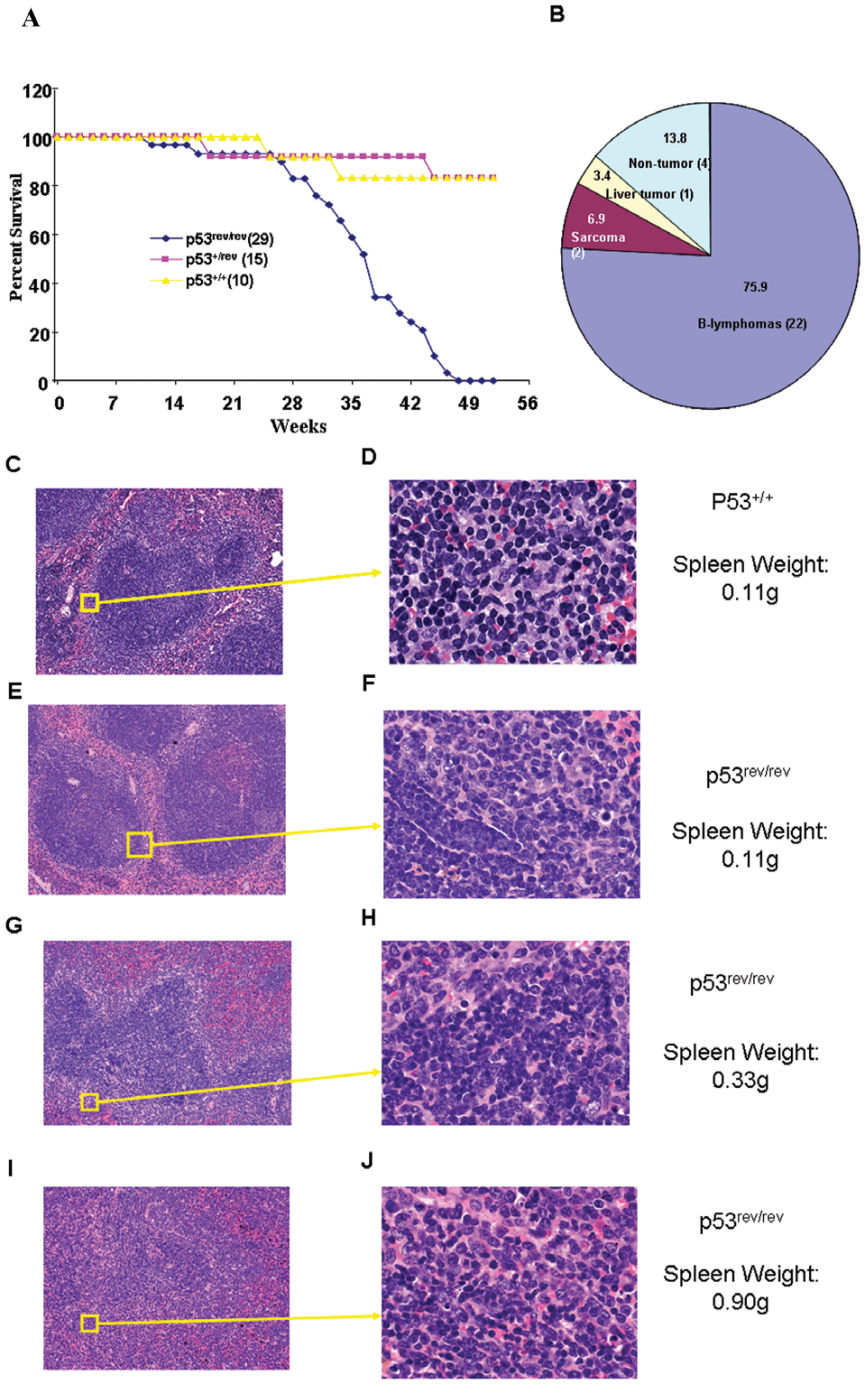
B cell lymphomas developed in p53^rev/rev^ mice. A) Survival of p53^rev/rev^ and control (p53^+/rev^ and p53^+/+^) mice. B) Pie chart shows percentages of cause of death for p53^rev/rev^ mice. C, E, G, I) Structure of splenic marginal zone for normal B6, normal p53^rev/rev^, and two individual tumor-bearing p53^rev/rev^ mice, respectively (H&E, 10×). D, F, H, J) High magnification of red pulp of and marginal zone for the spleen of a normal B6 mouse, normal p53^rev/rev^, and lymphoma-affected spleens from two different p53^rev/rev^ mice, respectively (H&E, 63×).

p53^−/−^ mice appear to develop normally, but spontaneously develop tumors by 6 months of age, approximately 75% of which are thymic T cell lymphomas [9]. To determine the effects of p53^rev^ expression on tumor development, we studied the survival of wt, p53^+/rev^ and p53^rev/rev^ mice for one year. The survival of both wt and p53^+/rev^ mice was 85% at one year with none of the mice dying of cancer (Figure 5A). In marked contrast, all p53^rev/rev^ mice were dead by 48 weeks of age (Figure 5A). Twenty-three of 29 p53^rev/rev^ mice had massive splenomegaly at the time of death. Histopathologic studies of 11 of these mice revealed changes characteristic of splenic marginal zone B cell lymphomas (SMZL) including marked expansion of the marginal zone with invasion of the red pulp, often associated with compression of the white pulp (Figure 5C–J). The lymphomas were limited to spleen except for one case with a subcutaneous metastasis. Of the remaining p53^rev/rev^ mice, two died with skin lesions, one with hepatomegaly, two with subcutaneous sarcomas, and two from unknown causes (Figure 5B).

**Figure 6:**
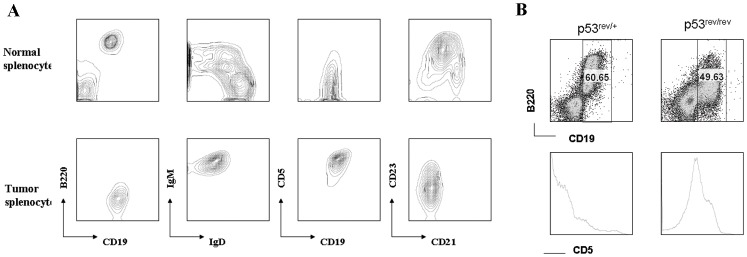
FACS analysis of cell surface markers of B cell lymphomas of p53^rev/rev^ mouse. A) Spleen cells of p53^+/+^ mouse (upper row) and B cell lymphomas of p53^rev/rev^ mice (bottom row) were stained with B220, IgM, IgD, CD21, CD23, CD19 and CD5 antibodies, and analyzed by FACS. B) Peripheral blood lymphocytes were isolated from p53^+/rev^ and p53^rev/rev^ mice, stained with B220, CD19 and CD5 antibodies, and analyzed by FACS.

**Figure 7:**
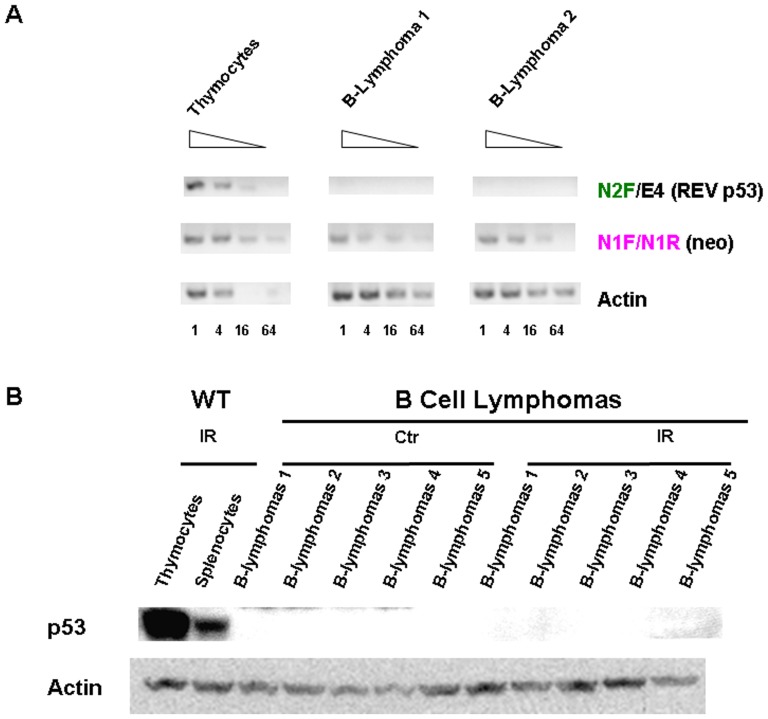
B cell lymphomas of p53^rev/rev^ mice did not express p53REV mRNA and p53 protein. A) RT-PCR analysis for p53REV and neomycin mRNA expression in B cell lymphomas. Actin was used as control. The results shown are representative of two independent experiments. B) Western blot analysis of p53 protein expression in six independent B cell lymphomas with (IR) or without (C) irradiation as indicated. Actin protein expression was used as loading control.

By flow cytometric analyses, the lymphoma cells were large and CD19^+^B220^lo^IgM^+^IgD^−^CD5^+^CD21^−^CD23^−^ (Fig. 6A). In tumor bearing mice, large B220^lo^CD5^+^ cells phenotypically similar to those of the splenic lymphoma could also be found in the peripheral blood (Fig. 6B) but not in the blood of young p53^rev/rev^ or wt mice. B cell lymphomas from p53^rev/rev^ mice did not express either p53REV mRNA or p53 protein, but were positive for expression of the neomycin resistance gene (Figure 7A, B).

Six B cell lymphomas from p53^rev/rev^ mice were subjected to karyotypic analysis (Table 1). Only one tumor was karyotypically normal (40, XY). The others exhibited a range of chromosome counts ranging from 2n to 4n and carried a variety of chromosomal anomalies including translocations, insertions and deletions. Four of the tumors were characterized by a single dominant clonal population, while two of the tumors were comprised of two dominant populations. Losses of chromosomes 12 and 19 and gains of chromosome 10 and 15 were often present. However, there was no chromosomal anomaly common to the different tumors that would suggest a unifying molecular mechanism. Of note, none of the B cell lymphomas analyzed had translocations involving Ig loci.

**Table 1:** SYK analysis of p53^rev/rev^ tumors.

Specimen	Sample Name	Genotype
B-Lymphoma	A1a	42–47, XX [2n] −1, +idel(2)(B;H3), idel(5)(B1;D), +der(5)T(5;15)(E;E),+10, −12, +15, Rb(16.idel(16)(B5 ;C2), +18, −19
		54–58, XXX [3n] −1, +idel(2)(B;H3), der(4)T(4;5)(E2;D), idel(5)(B1;D), +der(5)T(5;15)(E;E), −8, −9, +10, −12, +15, Rb(16.idel(16)(B5;C2), −19
B-Lymphoma	#4-082108A	40, XY [2n]
B-Lymphoma	#5-112008BL	40, XY [2n] +T(2;12), T(6;10), del(10), +10, −12, −12, +14, +15, dup(16)
B-Lymphoma	#7-112008BL	40,XX [2n] +T(2;12), T(6;10), T(10;17), −12, −12, +14, +15, −16
		75,XXXX [4n] +del(1), T(1;7), T(1;16), del(2), −3, T(3;7), del(4), +T(4;11), +T(4;X), −5, −5, −6, −8, T(8;1), −9, +10, T(10;17;4;8), T(11;3), T(12;3), T(13;3;….), −16, −16, −16, −18, +cen, +cen
B-Lymphoma	030209-8	58 X,- X,- X [3n] del(2)(C2), +del(2)(D), −5, −9, +10, +T(10;9)(C1;E1), −12, +T(15;2;15)(F3;C2 ->F1;??), −16, −19, +dmin(5)
B-Lymphoma	030209-13	82–91,XXXY [4n] T(2;3), T(3,2), +4, T(5;2), +10, +10, +10, +T(11;7), −12, +14, +15, +T(15;2)/T(15;5), +T(15;2)/T(15;5), −16, +17, +17, −19

### No B cell lymphomas developed in p53^rev/rev^ mice expressing B cell-specific CD19-Cre

**Figure 8:**
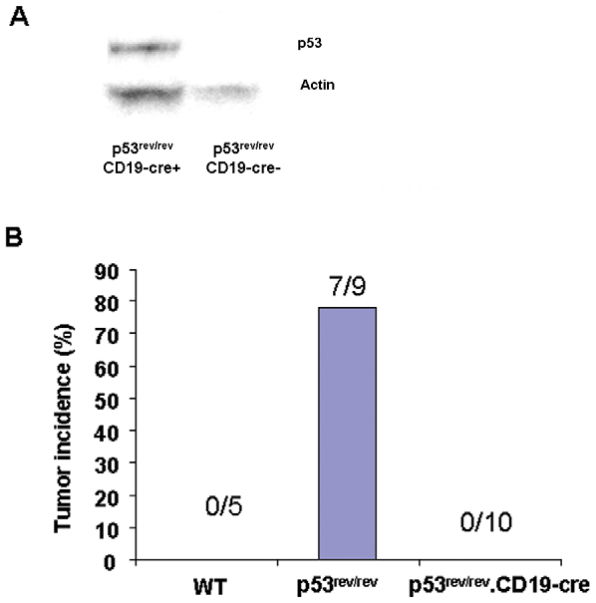
B cell lymphomas do not develop in p53^rev/rev^CD19-Cre^+^ mice. A) The B cells of p53^rev/rev^CD19-Cre^+^ and p53^rev/rev^CD19-Cre^−^ mice were isolated from splenocytes with MACS systems. The purified B cells were irradiated and cultured for three hours at 37°C. The lysates of B cells were immunoblotted with anti-p53 antibodies. B) B cell lymphoma incidence was determined in p53^rev/rev^CD19-Cre^+^ and p53^rev/rev^CD19-Cre^−^ mice followed for 9 months.

To determine whether p53 protein would be expressed in p53^rev/rev^ B cells in the presence of Cre expression, CD19-Cre transgenic mice [12] were bred with p53^rev/rev^ mice to generate p53^rev/rev^CD19-Cre^+^mice. Splenic B cells from p53^rev/rev^CD19-Cre^+^ and p53^rev/rev^CD19-Cre^−^ mice were isolated, irradiated and cultured for three hours at 37°C. The lysates of B cells were immunoblotted with p53 antibodies. Figure 8A shows that B cells from p53^rev/rev^CD19-cre^+^ mice were induced to express p53 protein while no detectable p53 protein was found in the B cells of p53^rev/rev^CD19-Cre^−^ mice. No B cell lymphomas were observed in ten p53^rev/rev^CD19-Cre^+^ mice followed for 9 months, while tumors developed in seven of nine p53^rev/rev^CD19-Cre^−^ mice (Figure 8B).

## Discussion

The tumor suppressor p53 plays a critical role in maintaining genomic integrity by regulating the expression of genes responsible for mediating apoptosis, cell cycle arrest, DNA repair, and senescence in response to DNA damage or oncogenic stress [3], [4]. More than 50% of human tumors harbor mutations in the p53 gene [3], [4], and essentially all p53^−/−^ mice develop tumors, predominantly thymic lymphomas, by one year of age [6], [9]. This study was designed to overcome the limitations on studying the contributions of p53 deficiency to transformation of cells other than T cells imposed by the early development of thymic lymphomas in conventional knockout mice. The approach was to generate mice, termed p53^rev/rev^ mice, in which expression of p53 was prevented by inserting a floxed neomycin resistance gene into the first exon of p53 gene, and in which cell lineage-specific p53 expression could be induced in the presence of an appropriate lineage-specific Cre. In contrast to these expectations, p53 protein was expressed in multiple tissues of p53^rev/rev^ mice, including thymus and spleen, in the absence of Cre. Studies of lymphocyte subsets from spleen showed that p53 was expressed by T cells but not B cells and that B cells remained negative for p53 expression, even following induction of DNA damage by irradiation. Strikingly, these mice developed splenic B cell lymphomas but no T cell lymphomas. Precedent for a role of p53 deficiency in B cell transformation exists in earlier studies of conventional p53^−/−^ mice that were shown to develop MZL at low frequency in the same time frame as thymic T cell lymphomas [22]. Interestingly, studies of human SMZL identified deletions and/or mutations in TRP53 in ∼20% of cases [23], [24]. Although these changes had no prognostic significance, they may indicate shared cross-species mechanisms in the pathogenesis of these poorly understood neoplasms. Interestingly, we had also been unsuccessful in efforts to make another reversible knockout mouse by using a neomycin resistance gene with loxP sites flanking SV40 polyA signal sequence as a STOP cassette. As shown in Figure S3, p53 expression was reduced by 90% but was not completely abolished when this STOP cassette was inserted into intron 6 of p53 gene. Moreover, this level of expression was sufficient to prevent appearance of tumors in p53^rk1/rk1^ mice.

The SMZL of p53^rev/rev^ mice were CD5^+^ and thus resembled a subset of human SMZL comprising about 25% of cases [25]. It is currently not clear if the CD5^+^ cases of human SMZL represent a phenotypic variant of SMZL with aberrant expression of CD5 or if they represent a separate clinico-pathologic entity. In mice, there are several models with lymphomas diagnosed histologically as SMZL that are CD5^+^ and may derive from peritoneal B1a cells rather than splenic MZ B cells [26], [27], [28]. Distinguishing between MZ and B1a cells as possible origins for these lymphomas will require the identification of reliable specific markers for each lineage that can be used for studies of normal and tumor cells.

The B cell lymphomas of p53^rev/rev^ mice were clearly different from the pro-B cell lymphomas observed in non-homologous end joining (NHEJ)-deficient and H2AX-deficient mice crossed onto a p53^−/−^ background, which are characterized by the presence of T(12;15) translocations [29], [30], [31], [32]. These translocations result in the juxtaposition of the *Myc* oncogene on chromosome 15 under the transcriptional regulation of the IgH promoter on chromosome 12. Interestingly, half of the karyotyped p53^rev/rev^ tumors had translocations involving chromosome 15 and 2/3 had an extra copy of chromosome 15, similar to what is observed in mouse thymic lymphomas.

By achieving altered cell lineage specificity of p53 expression, p53^rev/rev^ mice have created a novel and instructive model of B cell neoplasia. However, the regulatory mechanisms underlying this lineage-specific change in expression of p53 remain less than fully understood. We identified a transcriptional start site for p53REV mRNA located near the 3′ end of the neomycin resistance cassette that was utilized in thymocytes but not in B cells or B cell lymphomas of p53^rev/rev^ mice even though the neo gene was expressed at equal levels in these populations. This indicated that expression of p53REV mRNA was not determined simply by a foreign neo promoter. It thus seems likely that insertion of the neomycin gene in exon1 may disrupt the tissue specificity of an alternative p53 promoter, silencing the expression in B cells of p53^rev/rev^ mice. In initial experiments designed to further probe regulation of p53, we deleted the immediate promoter and partial first exon of the p53 gene in BAC DNA which was introduced as a transgene into p53^−/−^ mice. Surprisingly again, p53 protein was expressed in both thymocytes and splenocytes (Figure S4). Analysis of cDNA by 5′-RACE demonstrated a transcriptional start site within exon 1 of the p53 gene that is not the classic (common) site but corresponds to a cDNA sequence previously entered in GENEBANK (access number: CJ049635). Our data suggest that the p53 gene might have an unknown promoter that can act at long range to regulate p53 expression, as has now been described for a number of genes.

It is worth noting that while p53 protein is absent from the entire B cell population in p53^rev/rev^ mice, the lymphomas that develop in these mice bear the unique histopathologic features of SMZL and are thus quite distinct from the B lymphomas recently reported to occur in B cell-specific p53 knockout mice [33]; in that strain, p53-deficient B lineage cells were generated by the activity of mb1-Cre on a floxed p53 allele. The tumors that developed in those mice all expressed CD43, a B lineage marker that is extinguished when normal B cells rearrange the kappa locus during maturation in the bone marrow, suggesting that they all derived from immature B cells. Consistent with an origin in immature or pro-B cells, those tumors expressed translocations involving Ig loci, suggesting aberrant V(D)J rearrangement or class switch recombination. In contrast, the B cell lymphomas derived in our studies from p53^rev/rev^ mice expressed surface IgM and did not contain translocations involving Ig loci, suggesting that these lymphomas arose after normal and successful V(D)J recombination. In this regard it is noteworthy that SMZL also develop in other models in which p53 function is compromised but at low frequencies [33], [34]. The basis for this differential susceptibility of marginal zone B cells to transformation in these different experimental settings remains to be determined. The preferential development of SMZL in p53^rev/rev^ mice might reflect the stage of B cell development at which p53 protein expression is terminated in cells of this lineage, rendering this subset exceptionally susceptible to transformation. Analyses of developing B lineage cells in the bone marrow and spleen will be required to approach this question.

Previous studies reported that p53 mutations are associated with multiple subtypes of human B lymphomas, including mucosa-associated lymphoid tissue (MALT) lymphomas of marginal zone B cell origin, mantle cell lymphomas, centrocyte-like lymphomas, follicular lymphomas and SMZL [34]. The p53^rev/rev^ mouse therefore provides a novel animal model for understanding the mechanisms involved in the pathogenesis of the subsets of human B cell non-Hodgkin lymphomas that originate in marginal zone B cells. Importantly, p53 can be re-expressed at wt levels in p53^rev/rev^ B cell lymphomas when Cre is activated, thus providing a unique system to understand the influence of p53 expression at different stages of B cell lymphoma development and progression. The B1a B cell-like surface markers of p53^rev/rev^ B lymphomas suggested B1a B cells as possible cells of origin for these lymphomas. Therefore, the p53^rev/rev^ mouse may be a useful model for studies of MZ and B1 B cell lymphomas. Although the (p53^rk1/rk1^) mouse of Supplementary 3 was an unsuccessful reversible p53 knockout mouse, expression of p53 protein in this line is less than 10% of that of wild-type mice in the absence of cre; and it may therefore be a valuable animal model for understanding the effect of suppressed p53 expression level in tumor development.

## Supporting Information

Figure S1 A Western blot analysis of p53 protein and actin expression in various tissues of wt and p53^rev/rev^ mice as indicated. The results shown are representative of three independent experiments. To assess p53 protein in p53^rev/rev^ and wild-type controls, mice were irradiated at 10 Gy to increase p53 protein levels, largely through post-translational stabilization. Three hours later, tissues from these mice were used to make protein lysates that were analyzed by Western blotting with p53-specific antibodies. In wt mice, p53 protein levels were induced to readily detectable levels in all tissues tested with the exception of brain. Contrary to expectations, p53 protein was also detected in multiple tissues of p53^rev/rev^ mice, but with a pattern of tissue-specific expression that was substantially different from that of wt mice. p53 protein levels were similar to wt in thymus and lung but were markedly reduced in spleen, and uterus, and below the level of detection in liver.
(TIF)

Figure S2 A diagrammatic representation of the transcriptional start site of p53REV RNA. The pink box represents the neomycin resistance gene where the transcriptional start site was identified. The green arrow indicates the loxP site, the red box part of exon 1, and the blue box exon 2. Shown below is the DNA sequence, with different font colors matched with the colors of neomycin resistance gene, loxP sites, exon 1 and exon 2. 5′RACE products were cloned into TA vectors and 10 clones were sequenced. All clones identified only one species of 5′RACE product, designated p53REV.
(TIF)

Figure S3 A) To study the mechanism of development of thymic lymphomas in p53^−/−^ mice and to establish models of other p53-deficient tumors, another version of p53 reversible knockout mouse (p53^rk1/rk1^) was generated by the gene targeting. The neomycin resistance gene with SV40 polyA signal sequences flanked by loxP sites, which was the same as that used to generate the p53^rev/rev^ mouse, , was inserted into intron 6 of the p53 gene. It was expected that expression of p53 protein would be abolished in p53^rk1/rk1^ mice in the absence of cre. B) Western blot analysis of p53 protein expression in thymocytes and splenocytes of p53^+/+^ and p53^rk1/rk1^ mice without (C) or with (IR) irradiation as indicated. Actin protein expression was used as loading control. The results shown are representative of three independent experiments. It was unexpected that detectable levels of p53 protein were detected in both thymocytes and splenocytes of p53^rk1/rk1^ mouse. In addition, ten p53^rk1/rk1^ mice were observed for 18 months, with no tumors detected.
(TIF)

Figure S4 To assess the requirement for an immediate upstream p53 promoter in p53 expression, a BAC DNA, including the mouse p53 gene with at least 20 kb upstream and 20 kb downstream sequences, was mutated and used to make transgenic mice. A) The wild-type p53 gene with a HindIII restriction site inserted in intron 5 to allow distinguishing transgene and endogenous gene. The resulting BAC DNA was used to make control transgenic mice (p53Ctr). To delete the proximal 5′ promoter of p53, about 2 kb immediately upstream of and including a part of exon 1 of p53Ctr was deleted by BAC DNA engineering. The promoter-deleted BAC DNA designated as p53P1 was used to make mutant p53 transgenic mice. The p53Ctr and p53P1 transgenic mice were bred with p53^−/−^ mice to generate p53^−/−^p53Ctr and p53^−/−^p53P1 mice. It was striking that p53 protein could be detected in four independent lines of p53^−/−^p53P1 mice. B) Western blot analysis to determine p53 protein expression in thymocytes and splenocytes of p53^+/+^, p53^−/−^, p53^−/−^p53Ctr (2 lines) and p53^−/−^p53P1 (2 lines) mice following irradiation. Results shown are representative of 2 independent experiments. There was no consistent difference in the levels of p53 protein expression between p53^−/−^p53Ctr and p53^−/−^p53P1 mice. 5′-RACE was used to characterize the p53 mRNA in p53^−/−^p53P1 mice and revealed the transcriptional start site of p53 mRNA in exon 1 of p53 gene. This transcriptional start site is not the conventional start site but has previously been reported in GENEBANK (Access number: CJ049635).
(TIF)
