# Supplementary figures and images for: Exon 1 Disruption Alters Tissue-Specific Expression of Mouse p53 and Results in Selective Development of B Cell Lymphomas
Source: PLoS One. 2012 Nov 14;7(11):e49305. doi: 10.1371/journal.pone.0049305 (PMC3498120; doi:10.1371/journal.pone.0049305)

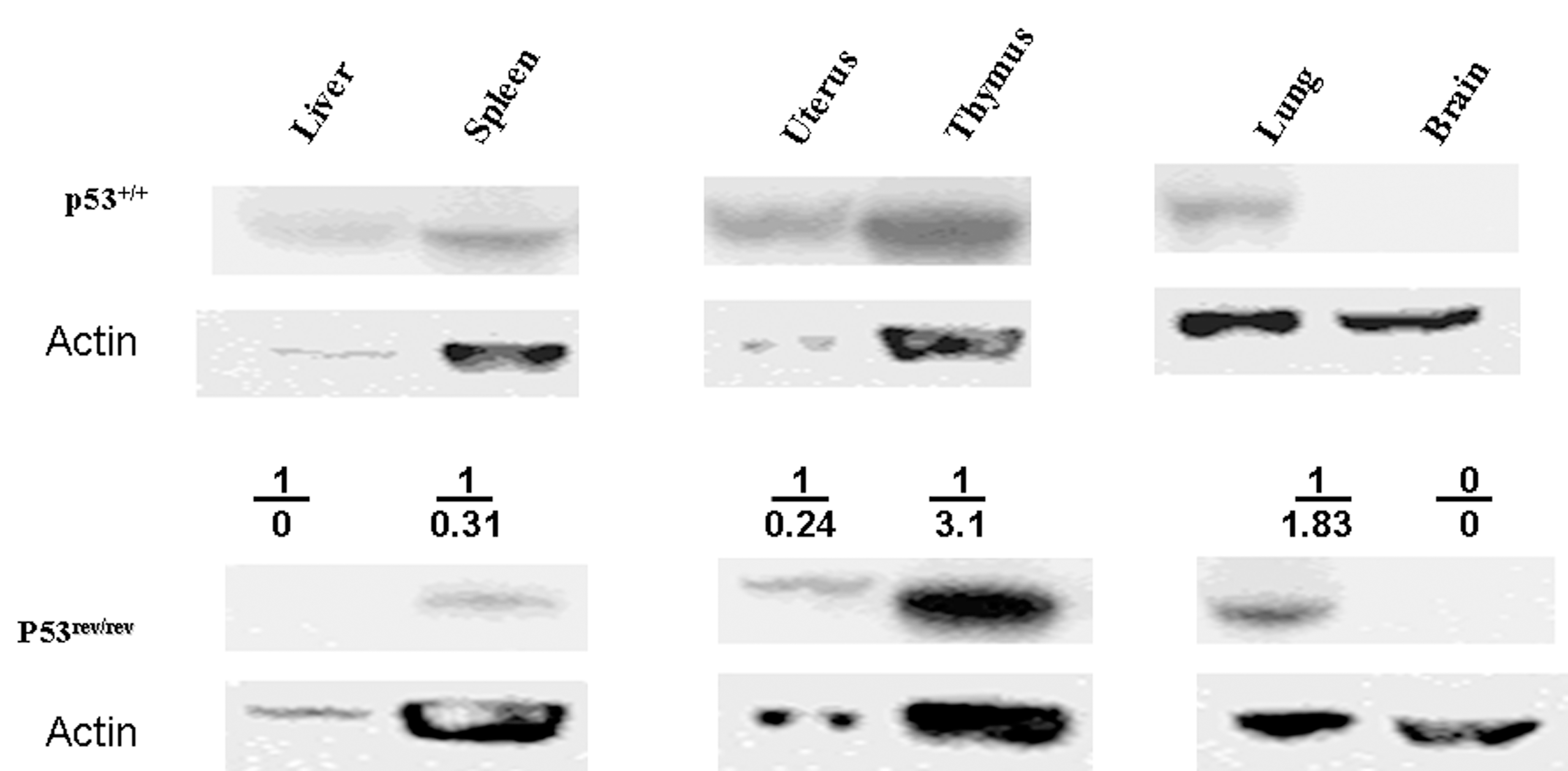

Supplement: Figure S1 — A Western blot analysis of p53 protein and actin expression in various tissues of wt and p53rev/rev mice as indicated. The results shown are representative of three independent experiments. To assess p53 protein in p53rev/rev and wild-type controls, mice were irradiated at 10 Gy to increase p53 protein levels, largely through post-translational stabilization. Three hours later, tissues from these mice were used to make protein lysates that were analyzed by Western blotting with p53-specific antibodies. In wt mice, p53 protein levels were induced to readily detectable levels in all tissues tested with the exception of brain. Contrary to expectations, p53 protein was also detected in multiple tissues of p53rev/rev mice, but with a pattern of tissue-specific expression that was substantially different from that of wt mice. p53 protein levels were similar to wt in thymus and lung but were markedly reduced in spleen, and uterus, and below the level of detection in liver. (TIF) [file pone.0049305.s001.tif]

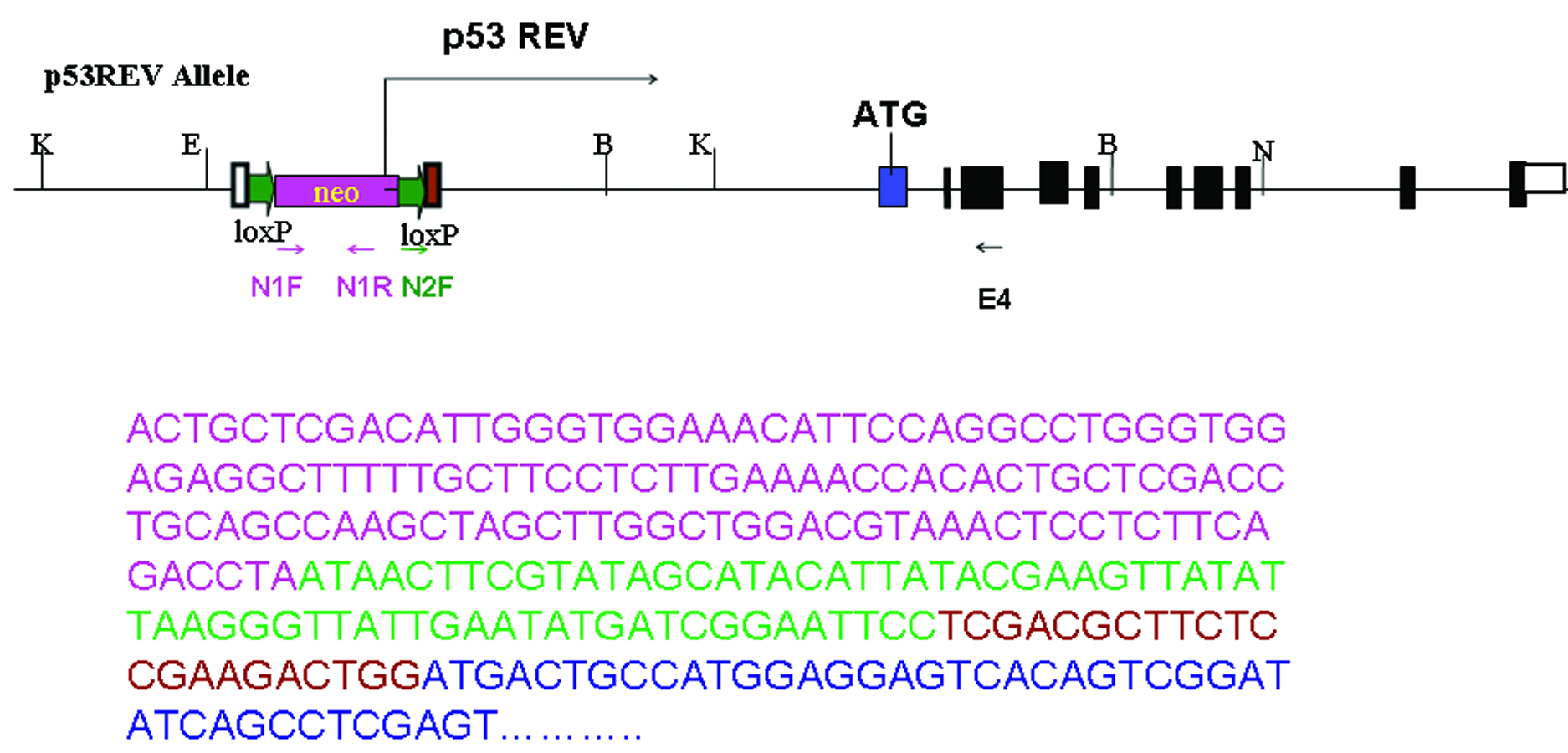

Supplement: Figure S2 — A diagrammatic representation of the transcriptional start site of p53REV RNA. The pink box represents the neomycin resistance gene where the transcriptional start site was identified. The green arrow indicates the loxP site, the red box part of exon 1, and the blue box exon 2. Shown below is the DNA sequence, with different font colors matched with the colors of neomycin resistance gene, loxP sites, exon 1 and exon 2. 5′RACE products were cloned into TA vectors and 10 clones were sequenced. All clones identified only one species of 5′RACE product, designated p53REV. (TIF) [file pone.0049305.s002.tif]

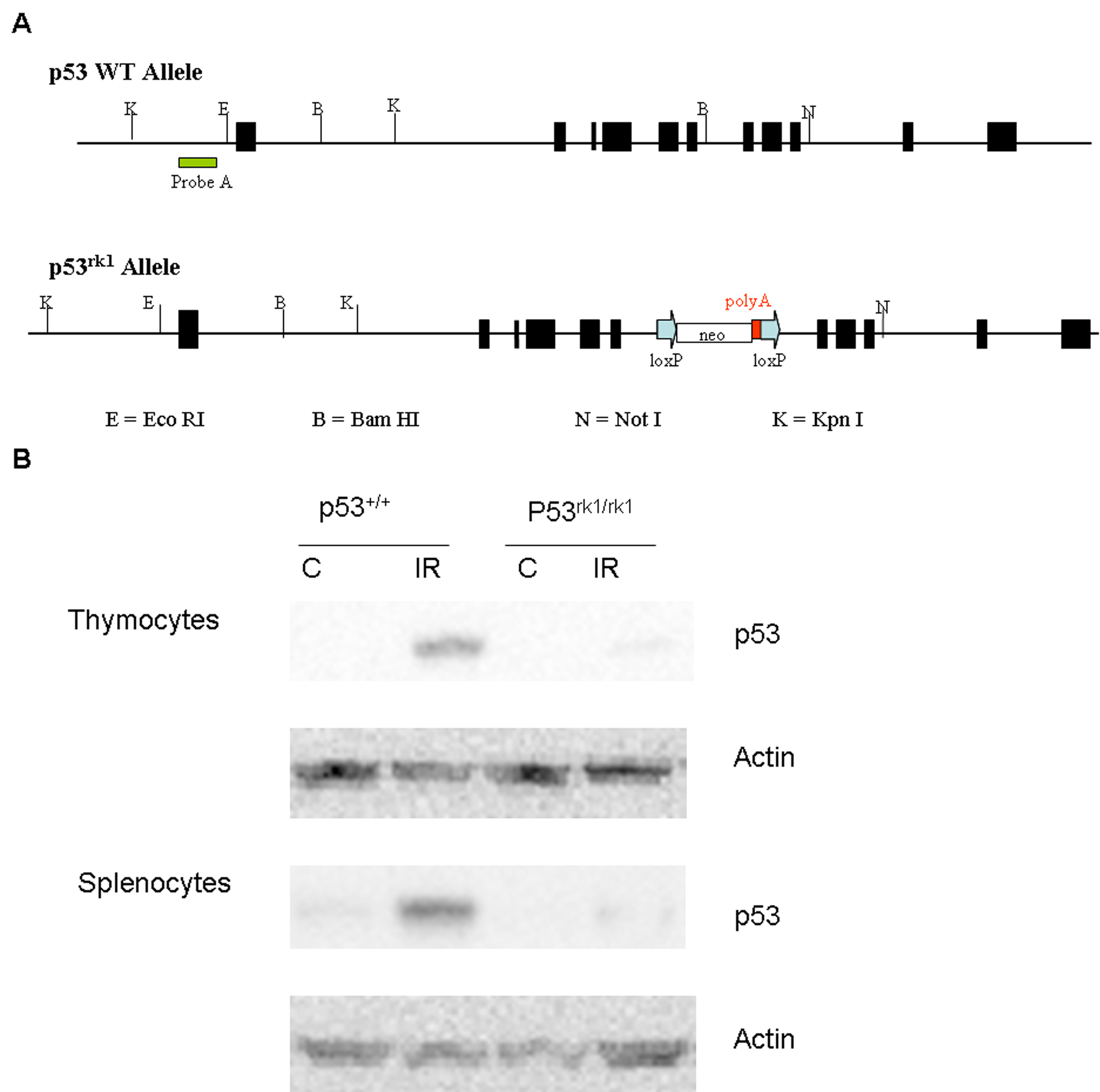

Supplement: Figure S3 — A) To study the mechanism of development of thymic lymphomas in p53−/− mice and to establish models of other p53-deficient tumors, another version of p53 reversible knockout mouse (p53rk1/rk1) was generated by the gene targeting. The neomycin resistance gene with SV40 polyA signal sequences flanked by loxP sites, which was the same as that used to generate the p53rev/rev mouse, , was inserted into intron 6 of the p53 gene. It was expected that expression of p53 protein would be abolished in p53rk1/rk1 mice in the absence of cre. B) Western blot analysis of p53 protein expression in thymocytes and splenocytes of p53+/+ and p53rk1/rk1 mice without (C) or with (IR) irradiation as indicated. Actin protein expression was used as loading control. The results shown are representative of three independent experiments. It was unexpected that detectable levels of p53 protein were detected in both thymocytes and splenocytes of p53rk1/rk1 mouse. In addition, ten p53rk1/rk1 mice were observed for 18 months, with no tumors detected. (TIF) [file pone.0049305.s003.tif]

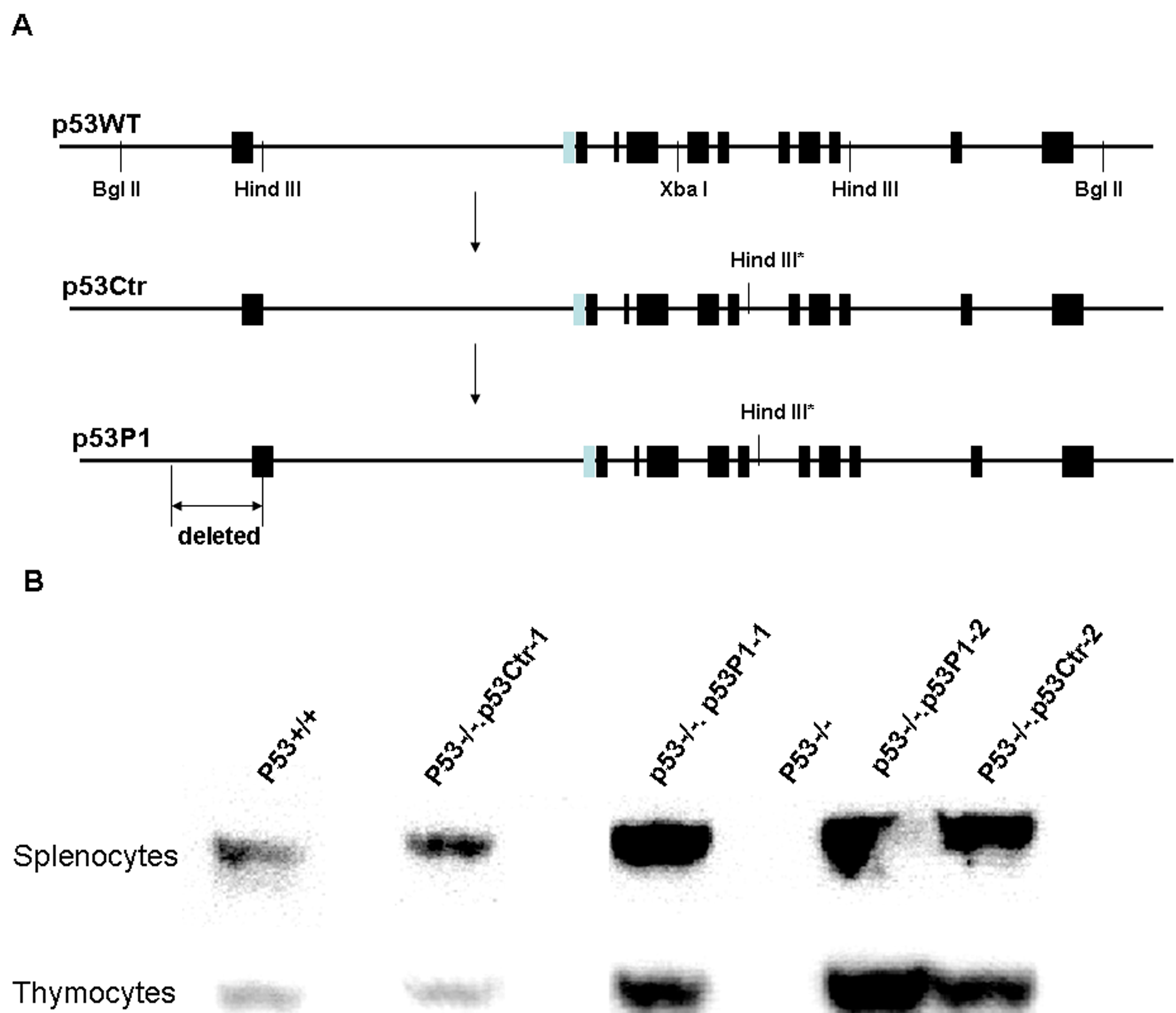

Supplement: Figure S4 — To assess the requirement for an immediate upstream p53 promoter in p53 expression, a BAC DNA, including the mouse p53 gene with at least 20 kb upstream and 20 kb downstream sequences, was mutated and used to make transgenic mice. A) The wild-type p53 gene with a HindIII restriction site inserted in intron 5 to allow distinguishing transgene and endogenous gene. The resulting BAC DNA was used to make control transgenic mice (p53Ctr). To delete the proximal 5′ promoter of p53, about 2 kb immediately upstream of and including a part of exon 1 of p53Ctr was deleted by BAC DNA engineering. The promoter-deleted BAC DNA designated as p53P1 was used to make mutant p53 transgenic mice. The p53Ctr and p53P1 transgenic mice were bred with p53−/− mice to generate p53−/−p53Ctr and p53−/−p53P1 mice. It was striking that p53 protein could be detected in four independent lines of p53−/−p53P1 mice. B) Western blot analysis to determine p53 protein expression in thymocytes and splenocytes of p53+/+, p53−/−, p53−/−p53Ctr (2 lines) and p53−/−p53P1 (2 lines) mice following irradiation. Results shown are representative of 2 independent experiments. There was no consistent difference in the levels of p53 protein expression between p53−/−p53Ctr and p53−/−p53P1 mice. 5′-RACE was used to characterize the p53 mRNA in p53−/−p53P1 mice and revealed the transcriptional start site of p53 mRNA in exon 1 of p53 gene. This transcriptional start site is not the conventional start site but has previously been reported in GENEBANK (Access number: CJ049635). (TIF) [file pone.0049305.s004.tif]
